# Mental distress in ongoing conflict and non-conflict settings during COVID-19: a study on Syrians in different countries

**DOI:** 10.1017/gmh.2021.12

**Published:** 2021-04-05

**Authors:** Ameer Kakaje, Ammar Fadel, Ayham Ghareeb, Ragheed Al Zohbi

**Affiliations:** 1 Faculty of Medicine, Damascus University, Damascus, Syria; 2 Faculty of Medicine, Aleppo University, Aleppo, Syria

Dear Editor,

Syria has suffered for 9 years from war which caused more than half of the children and adults to suffer from severe post-traumatic stress disorder (PTSD) and/or mental distress (Kakaje *et al*., 2020*a*, *b*). Even when leaving the country and being refugees, around 25% of people who left the country have suffered from post-traumatic stress symptoms (PTSS), depression, and/or anxiety (Georgiadou *et al*., 2018). This study evaluates the mental distress in Syrians who left the country because of war and in those who stayed in the conflict in Syria in the last years.

As self-reporting measures tend to overestimate symptoms, scores were used rather than cut-off points. Only Syrians were included, either living in Syria or those who left Syria due to war regardless of the method and Visa status and they were recruited using social media groups via online questionnaires. No participants living in camps were included. For each participant living outside Syria, a matching case living in Syria was randomly chosen. We only matched for age and gender as other variables would vary between the countries such as salaries and the socioeconomic status standards.

The Screen for Post-traumatic Stress Symptoms (SPTSS) tool was used for PTSS assessment which does not require a single traumatic event and screens for PTSS (Aljurany, 2013; Kakaje *et al*., 2020*a*). Multidimensional Scale of Perceived Social Support (MSPSS) for social support (R and Kazarian, 2012; Zimet, 2016) and Kessler 10 (K10) for anxiety and depression were also used (Andrews and Slade, 2001; Kessler *et al*., 2002; Palazón-Bru *et al*., 2018). These methods were validated in Arabic and previously used in the Syrian community (Kakaje *et al*., 2020*a*).

Income was assessed based on the income being adequate for needs rather than how much was earned (Kakaje *et al*., 2020*a*). Nearby countries category involved countries that surrounded Syria which were Lebanon, Jordan, Turkey, Iraq, and Iran. Data were processed using IBM SPSS software version 26 for Windows.

Due to the short time of data collecting and the urgency of COVID-19, a larger sample size was not achievable. Around 241 participants from outside Syria received the survey, but only 236 agreed to participate. A larger number of people agreed to participate from Syria. However, only one matching participant was randomly chosen whereas the others were excluded. This study included 472 participants, of which 214 (45.3%) were males with a mean age of 29.1 ± 7.5 years.

There were no significant differences in SPTSS, K10, and social support scores between different governorates within Syria (*p* > 0.05). Living in Syria was associated with a higher level of education, owning houses or apartments rather than tenants, being singles, and having less monthly income adequacy compared to living in other countries (*p* < 0.001). Results of the scales are demonstrated in Table 1. When applying cut-off points, Syrians in Syria comprised of 72 (31.3%) with avoidance symptoms, 106 (46.5%) with arousal symptoms, and 103 (45.4%) with re-experience symptoms, whereas Syrians outside Syria comprised of 96 (41.6%) with avoidance symptoms, 112 (49.6%) with arousal symptoms, and 111 (50.5%) with re-experience symptoms. Therefore, only avoidance was significantly more frequent in Syria (*p* = 0.022).

**Table 1.** K10 scores, days unable to work, social support and PTSD in different countries

Characteristic	Syria mean (s.d.)	Outside Syria mean (s.d.)	*p* value	Nearby country mean (s.d.)	Canada mean (s.d.)	Egypt mean (s.d.)	UAE mean (s.d.)	Russia mean (s.d.)	Germany mean (s.d.)	*p* value^a^
Arousal	1.55 (1.427)	1.79 (1.563)	0.089	1.80 (1.560)	1.46 (1.266)	1.69 (1.569)	1.96 (1.641)	2.73 (1.272)	1.56 (1.586)	0.131
Avoidance	1.82 (1.726)	2.17 (1.889)	0.035	2.59 (1.968)	1.69 (1.974)	2.04 (1.605)	2.17 (1.872)	2.00 (2.145)	1.82 (1.820)	0.067
Re-experience	0.93 (1.284)	1.21 (1.600)	0.042	1.38 (1.686)	1.00 (1.206)	0.85 (1.287)	1.27 (1.724)	1.18 (1.471)	1.15 (1.638)	0.307
Total SPTSS	4.30 (3.735)	5.07 (4.422)	0.045	5.71 (4.630)	4.08 (3.796)	4.48 (3.435)	5.27 (4.698)	5.91 (4.571)	4.39 (4.422)	0.158
Friends support	15.620 (7.5494)	15.232 (7.7590)	0.590	15.372 (8.1975)	14.269 (8.9550)	15.115 (6.4750)	16.848 (8.0415)	7.727 (4.5131)	15.481 (7.0557)	0.038
Family support	19.097 (6.9193)	19.613 (6.8274)	0.423	18.633 (6.9157)	19.500 (9.0692)	19.058 (6.5793)	20.924 (6.6533)	20.500 (7.5299)	19.972 (6.2935)	0.632
Significant other support	19.273 (7.6526)	20.226 (7.3404)	0.174	19.880 (6.8556)	19.923 (8.4307)	19.111 (8.1090)	21.340 (7.4324)	18.864 (7.7495)	20.636 (7.3500)	0.653
Total social support	53.85 (18.307)	54.55 (18.263)	0.686	53.68 (17.675)	53.69 (21.092)	52.02 (19.812)	58.31 (19.702)	47.09 (15.152)	55.45 (16.854)	0.567
Total K 10 support	22.5633 (8.74049)	23.4474 (9.63162)	0.305	23.8108 (9.26131)	20.1538 (10.91517)	24.6071 (10.71288)	24.4681 (8.74481)	20.3636 (7.24255)	22.8909 (10.42375)	0.508
Days unable to work in the past 4 weeks	7.81 (11.050)	179 220.15 (2 444 117.919)	0.326	584 802.67 (4 415 106.822)	5.55 (9.048)	5.41 (6.681)	14.82 (58.913)	7.44 (7.502)	7.88 (7.860)	0.493

We used one-way analysis of variance and independent *t* test in this table.

a This *p* value is when comparing Syria with the previous countries.

Doctors and dentists living in Syria had higher scores than the others compared to living in other countries.

Participants from Syria were more worried from the reduced ability to earn money, being unable to provide food, and their relationship with their deteriorated friends or family (*p* < 0.05), but they were less worried about getting infected (*p* = 0.041) compared with Syrians outside. However, there were no significant differences in the relationship with the partner and housemates, being distressed from war noise, being themselves or family exposed/infected to COVID-19, being hospitalized, self-quarantined with or without symptoms, losing jobs, and worries of work and study being affected. K10 score was not statistically significantly different according to the type of work.

COVID-19 outbreak was associated with higher PTSS for Syrians outside Syria, which might be from Syria having fewer cases and being more stable socially as they lived with their families in their houses, not in a foreign country. This could also be from the 4-week lockdown providing a break for people, which helped in decreasing their symptoms in Syria.

New methods should be introduced to target Syrian populations as up-to-date and accurate data can be protective against mental distress (Alyousbashi and Almahayni (n.d); Luo *et al*., 2020). This is particularly prominent in societies such as Syria as the majority of people are daily users of social media and therefore it will provide an easy and fast way to reach the population. The majority of Syrians get their information from social media which leaves a large window of false information. It was difficult to reach the targeted population in our study, so online surveys in social media groups were used which may expose to bias and might have made it more difficult to compare with other samples. However, social media has proven to be a viable item for global mental health which allowed us to reach populations, easier and faster, but we still need to develop the tools to tackle this multi-dynamic crisis. This also justifies the high response rate as these are people who had time and the surveys were easy to fill in, which might have also biased the results as the people who did not have time or Internet access would not have seen the surveys.

In conclusion, as Syria did not have many active cases and the conflict was relatively less severe at the time of this study, Syrians in Syria were particularly concerned about providing food and a reduced ability to earn. This study suggests that Syrians who had to leave and live in a foreign country where COVID-19 outbreak started to rise faced a different mental distress than those who stayed during the war despite both groups having roughly the same social support. This study suggests that mental issues inside Syria are mostly from the deteriorating economy whereas for Syrians outside suffered mostly from COVID-19. In a time of accelerating, ongoing, dynamically overlapping, and rapidly evolving global crises such as COVID-19, more nimble and versatile ways of tracking mental health change longitudinally and comparatively are needed.

## Data Availability

The data can be made available upon a reasonable request.
